# Backward simulation for inferring hidden biomolecular kinetic profiles

**DOI:** 10.1016/j.xpro.2021.100958

**Published:** 2021-11-15

**Authors:** Junghun Chae, Roktaek Lim, Cheol-Min Ghim, Pan-Jun Kim

**Affiliations:** 1 Department of Physics, Ulsan National Institute of Science and Technology, Ulsan 44919, Republic of Korea; 2 Department of Biology, Hong Kong Baptist University, Kowloon, Hong Kong; 3 Department of Biomedical Engineering, Ulsan National Institute of Science and Technology, Ulsan 44919, Republic of Korea; 4 Center for Quantitative Systems Biology & Institute of Computational and Theoretical Studies, Hong Kong Baptist University, Kowloon, Hong Kong; 5 State Key Laboratory of Environmental and Biological Analysis, Hong Kong Baptist University, Kowloon, Hong Kong; 6 Abdus Salam International Centre for Theoretical Physics, 34151 Trieste, Italy

**Keywords:** Biophysics, Systems biology, Computer sciences

## Abstract

Our backward simulation (BS) is an approach to infer the dynamics of individual components in ordinary differential equation (ODE) models, given the information on relatively downstream components or their sums. Here, we demonstrate the use of BS to infer protein synthesis rates with a given profile of protein concentrations over time in a circadian system. This protocol can also be applied to a wide range of problems with undetermined dynamics at the upstream levels.

For complete details on the use and execution of this protocol, please refer to Lim et al. (2021).

## Highlights

• Inference of upstream dynamics with information on the downstream profile
• Applies to infer protein synthesis rates with a given circadian protein profile
• Widely applicable to systems biology models with known downstream profiles

## Graphical abstract

**Figure:**
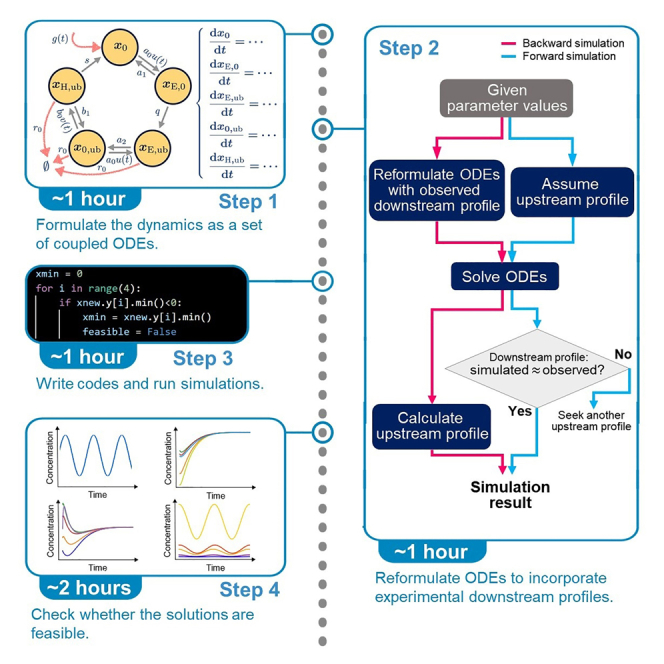

## Before you begin

### Check whether the backward simulation (BS) is desirable

**Timing: 10 min**

We have developed the backward simulation (BS) method to discover the “internal” dynamics of the system described by ordinary differential equations (ODEs) with pre-selected model structure and parameter values*.* When the temporal profiles of relatively downstream components or their sums (but not the profiles of upstream components) are known, BS retrieves the upstream profiles that exactly reproduce the known downstream profiles, through the straightforward ODE calculation with the relevant downstream variables. On the other hand, an existing practice is to assume the plausible forms of these unknown upstream profiles with additional free parameters, and then estimate these parameters to best fit the observed downstream profiles. However, in contrast to the BS, the latter method incurs the computational costs for that parameter estimation and may not even necessarily reproduce the correct downstream profiles.

As a prototype application of this method, we here elaborate the case of the circadian protein degradation model in Lim et al. (2021). Specifically, in contrast to a common practice of simulating the time-course profile of a circadian protein concentration by its upstream elements such as the rhythmic synthesis rate of the protein over time, we intended to maintain the total protein concentration profile at the downstream side as it was and simulate the corresponding rate of the protein synthesis and other upstream kinetic processes in given parameter conditions (Lim et al., 2021). There were three main reasons for this simulation: (*i*) unlike the mRNA profile, the potentially time-of-day-specific translation rate per mRNA is not commonly known and hence it is difficult to determine the profile of the protein synthesis rate directly from the existing experimental data. In contrast, the experimental profile of the total protein levels is readily available for the incorporation to model simulations. In Lim et al., 2021, given the experimental protein profiles, we performed the BS over randomly-sampled parameter values, and identified the parameter sets for simulation results in quantitative agreement with the empirically observed, rhythmic degradation rates of the proteins. (*ii*) Another reason was that we wanted to dissect the underpinning mechanism of the rhythmic degradation rate of a circadian protein by *rigorously controlling for the effect of the protein profile* over a range of parameter values, while avoiding the confounding effect from the changes in the protein profile itself caused by the conventional simulation with given profiles of upstream elements (“forward simulation”). (*iii*) Lastly, we considered an evolutionary viewpoint that the protein profile can be of a more fundamental position than an mRNA or translation-rate profile so that the protein synthesis rate may have adapted to the protein profile—the protein profile is more likely to influence a biological phenotype than other elements in the system such as mRNA profile. Overall, we foresee a variety of applications of the BS, including the cases with data unavailability at upstream sides, the mechanistic studies with strictly-controlled downstream profiles, and the evolutionary modeling with fixed downstream profiles.

### Install python and python packages

**Timing: 10 min**

1. Download Python 3.7.4 or a higher version from https://www.python.org. The Python version can be checked by the following command:

> python3 –version

2. To solve ODE models, the scipy python module is needed. Install scipy package:

> pip install scipy

## Key resources table

**Table:** 

REAGENT or RESOURCE	SOURCE	IDENTIFIER
**Software and algorithms**		

Custom codes for model simulation	This paper	https://git.io/JuAFt
Python 3.7.4 or higher version	Python Software Foundation	https://www.python.org
SciPy v1.3.1 or higher version	Virtanen et al. (2020)	https://www.scipy.org

## Step-by-step method details

### BS in general cases

**Timing: 1 h for step 1 and 1 h for step 2**

In this section, we will describe how to apply the BS to a general system of ODEs where the downstream profile is given.

1. Formulate the dynamics as a set of coupled ODEs.
  a. For a dynamical system with *n* variables $\left[y_{1}\left(t\right),y_{2}\left(t\right),\cdots ,y_{n}\left(t\right)\right]\equiv \vec{y}\left(t\right)$ and *m* parameters $\left[p_{1}\left(t\right),p_{2}\left(t\right),\cdots ,p_{m}\left(t\right)\right]\equiv \vec{p}\left(t\right)$ that can be described by a set of coupled ODEs, we divide the rate processes into two parts, i.e.,
    (Equation 1) $$\frac{dy_{i}\left(t\right)}{dt}=F_{i}\left[\vec{p}\left(t\right);\vec{y}\left(t\right)\right]+G_{i}\left[\vec{p}\left(t\right);\vec{y}\left(t\right),t\right],i=1,2,\cdots ,n,$$
    where $F_{i}\left[\vec{p}\left(t\right);\vec{y}\left(t\right)\right]$describes the intermediate or interconversion processes of components satisfying $\overset{n}{\underset{i=1}{\sum }}F_{i}\left[\vec{p}\left(t\right);\vec{y}\left(t\right)\right]=0$, and $G_{i}\left[\vec{p}\left(t\right);\vec{y}\left(t\right),t\right]$ describes the source/sink-coupled, nonconservative events responsible for the changes in the total pool of *y*_*i*_(*t*) (*i*=1,2,⋯,*n*).
  b. Summing up the left- and right-hand sides of Equation 1 over *i*, all the terms responsible for intermediate processes cancel out, leaving only the source/sink-coupled terms:
    $$\frac{d}{dt}\overset{n}{\underset{i=1}{\sum }}y_{i}\left(t\right)=\overset{n}{\underset{i=1}{\sum }}G_{i}\left[\vec{p}\left(t\right);\vec{y}\left(t\right),t\right].$$
  c. Take extra care of any fundamental conditions of the variables and terms in the model. For example, if the variables represent molecular concentrations, they should be nonnegative, i.e., *y*_*i*_(*t*)≥0 for *i*=1,2,⋯,*n*. Likewise, a nonnegative net influx of component *y*_*j*_(*t*) from an external source indicates $G_{j}\left[\vec{p}\left(t\right);\vec{y}\left(t\right),t\right]\geq 0$.

2. Transform the ODEs to utilize the accessible information on relatively downstream variables in the ODE model.
  a. Introduce an observable time-course variable *Y*(*t*) as $Y\left(t\right)\equiv \underset{i\in A}{\sum }y_{i}\left(t\right)$ where *A* is the set of components whose sum is of an experimentally available quantity or of theoretical interest. For example
  b. For a particular component *j* selected among the elements of *A*, we rewrite *y*_*j*_(*t*) as
    (Equation 2) $$y_{j}\left(t\right)=Y\left(t\right)-\sum_{i\in A\backslash \left\{j\right\}}y_{i}\left(t\right).$$
    Then, we define ${\vec{y}}_{Y}\left(t\right)$ as an alternative form of $\vec{y}\left(t\right)$ where *y*_*j*_(*t*) is replaced by the right-hand side of Equation 2 and rewrite Equation 1 for i≠j:
    (Equation 3) $$\frac{dy_{i}\left(t\right)}{dt}=F_{i}\left[\vec{p}\left(t\right);{\vec{y}}_{Y}\left(t\right)\right]+G_{i}\left[\vec{p}\left(t\right);{\vec{y}}_{Y}\left(t\right),t\right],i\neq j.$$
  c. Combining Equations 2 and 3 for *i*=*j*, we obtain
    (Equation 4) $$G_{j}\left[\vec{p}\left(t\right);{\vec{y}}_{Y}\left(t\right),t\right]=\frac{dY\left(t\right)}{dt}-\sum_{k\in A\backslash \left\{j\right\}}\frac{dy_{k}\left(t\right)}{dt}-F_{j}\left[\vec{p}\left(t\right);{\vec{y}}_{Y}\left(t\right)\right].$$
    The conventional forward simulation numerically solves Equation 1 to obtain $\vec{y}\left(t\right)$, given the values of $\vec{p}\left(t\right)$ and $G_{i}\left[\vec{p}\left(t\right);\vec{y}\left(t\right),t\right]$ and the initial condition of $\vec{y}\left(t\right)$. On the other hand, our BS numerically solves Equations 3 and 4 to obtain $G_{j}\left[\vec{p}\left(t\right);{\vec{y}}_{Y}\left(t\right),t\right]$ and ${\vec{y}}_{Y}\left(t\right)$, given the values of $\vec{p}\left(t\right)$, *Y*(*t*), and $G_{i}\left[\vec{p}\left(t\right);{\vec{y}}_{Y}\left(t\right),t\right]$ (i≠j) and the initial condition of ${\vec{y}}_{Y}\left(t\right)$. In other words, using a downstream observable *Y*(*t*), BS traces back the upstream processes such as $G_{j}\left[\vec{p}\left(t\right);{\vec{y}}_{Y}\left(t\right),t\right]$.
  d. If the computed $G_{j}\left[\vec{p}\left(t\right);{\vec{y}}_{Y}\left(t\right),t\right]$ or ${\vec{y}}_{Y}\left(t\right)$ does not satisfy the fundamental conditions imposed by a modeler, they are treated as infeasible solutions. For example, if *y*_*i*_(*t*) in ${\vec{y}}_{Y}\left(t\right)$represents the concentration of each molecular species *i*, *y*_*i*_(*t*) should be non-negative for all *i*’s. Depending on cases, the infeasible solutions may indicate the incompatibility of a simulated parameter set $\vec{p}\left(t\right)$ to an observed profile of *Y*(*t*).

### Application of BS to a circadian protein degradation model

**Timing: 1 h for step 3, 1 h for step 4, 1 h for step 5, and 2 h for step 6**

In this section, we will describe how to apply the BS to a circadian protein degradation model in Lim et al. (2021). Step 3 and 4 show how to modify the set of coupled ODEs of a circadian protein degradation model for the BS. How to write the codes for the BS and how to examine the ODE system are included in step 5 and 6, respectively.

**Figure 1:**
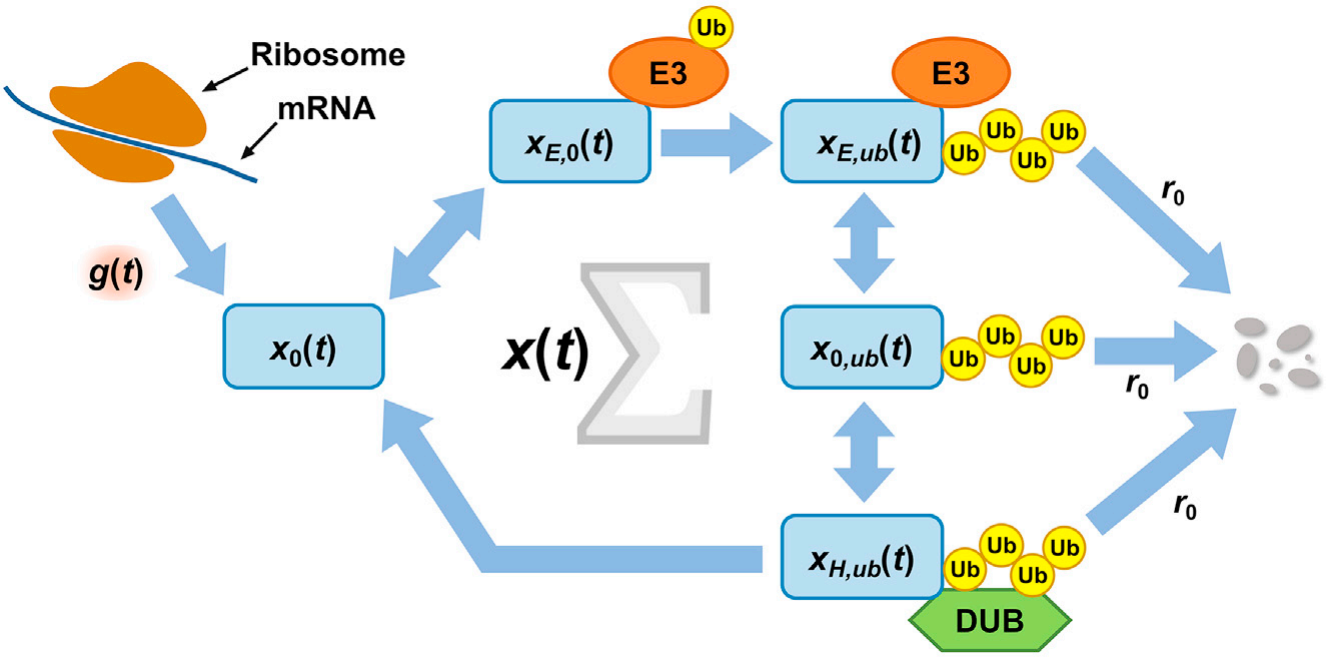
The protein degradation model There are a total of five types of substrate proteins, defined in Table 2. Substrate proteins (rounded rectangles, sky blue) are synthesized from mRNA molecules (blue line, top left) in the ribosome (brown, top left) and ubiquitinated by E3 ubiquitin ligases (orange ovals) with ubiquitins (yellow circles). The ubiquitinated proteins are degraded (gray ovals) or deubiquitinated by deubiquitinating enzymes (light green hexagon). The total protein concentration is represented by *x*(*t*) with symbol *Σ* at the center.

**Figure 2:**
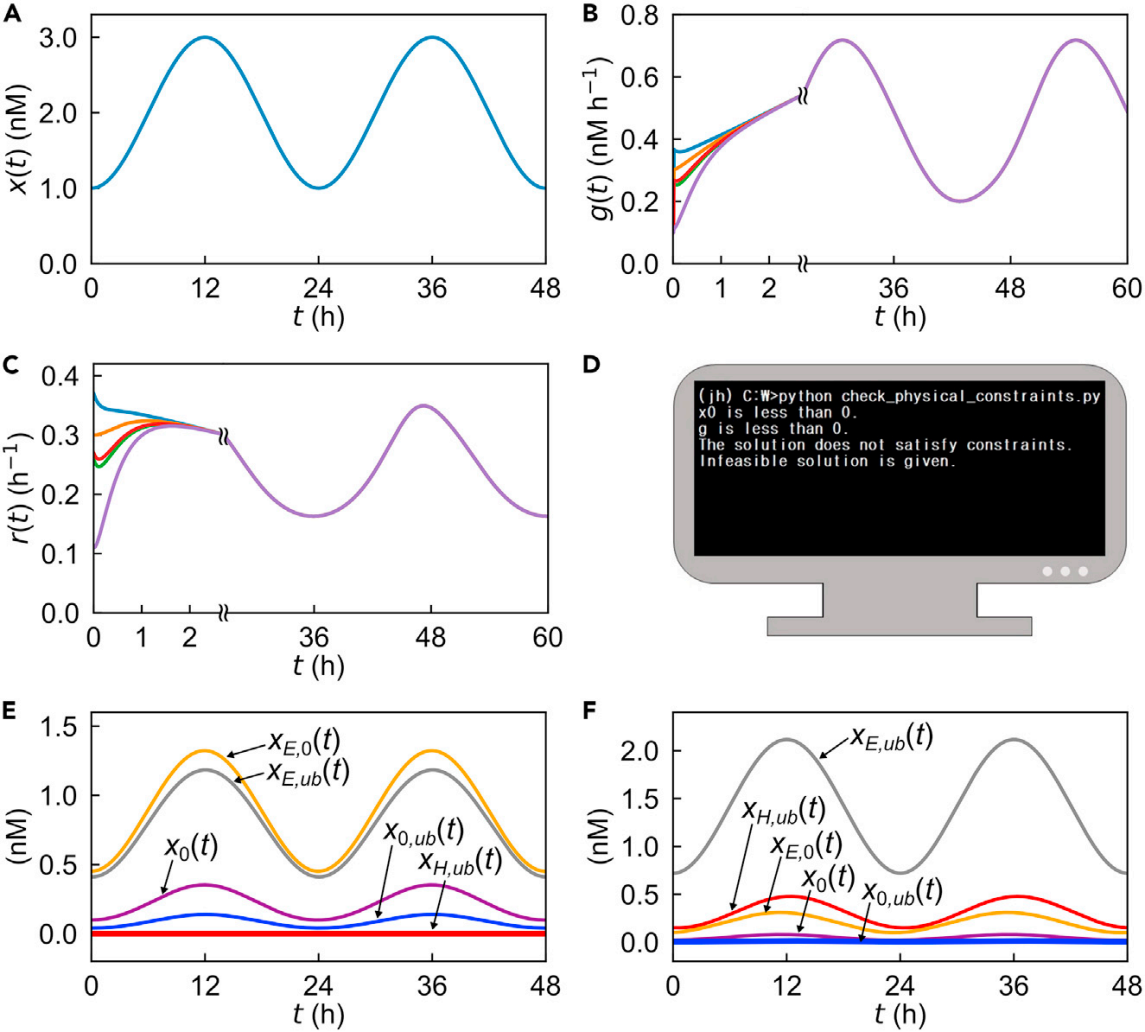
Examining the behaviors of the protein degradation model (A) The example, total protein concentration profile used for BS. (B and C) Simulated *g*(*t*) (B) and *r*(*t*) (C) with different initial conditions. Given the protein profile in (A), the simulated *g*(*t*) or *r*(*t*) rapidly converges at the same trajectory, regardless of its initial conditions. (D) The example output of the code “check_physical_constraints.py” to check the feasibility of the BS solution. If the solution does not satisfy its fundamental conditions, the code returns the message in (D). (E and F) Simulated profiles of protein sub-states: *x*_0_(*t*) (purple), *x*_*E*,0_(*t*) (yellow), *x*_*E*,*ub*_(*t*) (gray), *x*_0,*ub*_(*t*) (blue), and *x*_*H*,*ub*_(*t*) (red). When $\bar{v}$′ is set to zero, *x*_*H*,*ub*_(*t*) becomes zero (E). When *a*_0_, *b*_0_, $\bar{u}$′, and $\bar{v}$′ are set as relatively high and *a*_2_ and *b*_1_ are set as relatively low, *x*_0,*ub*_(*t*) becomes almost zero (F).

3. Formulate the dynamics as a set of coupled ODEs.
  a. In our circadian protein degradation model in Lim et al. (2021), the time derivatives of the concentrations of several forms of a circadian protein are described as follows (Figure 1):
    (Equation 5) $$\frac{dx_{0}\left(t\right)}{dt}=g\left(t\right)-a_{0}u\left(t\right)x_{0}\left(t\right)+a_{1}x_{E,0}\left(t\right)+sx_{H,ub}\left(t\right),$$
    (Equation 6) $$\frac{dx_{E,0}\left(t\right)}{dt}=a_{0}u\left(t\right)x_{0}\left(t\right)-a_{1}x_{E,0}\left(t\right)-qx_{E,0}\left(t\right),$$
    (Equation 7) $$\frac{dx_{E,ub}\left(t\right)}{dt}=qx_{E,0}\left(t\right)+a_{0}u\left(t\right)x_{0,ub}\left(t\right)-a_{2}x_{E,ub}\left(t\right)-r_{0}x_{E,ub}\left(t\right),$$
    (Equation 8) $$\begin{array}{c} \;\frac{dx_{0,ub}\left(t\right)}{dt}=a_{2}x_{E,ub}\left(t\right)+b_{1}x_{H,ub}\left(t\right)-b_{0}v\left(t\right)x_{0,ub}\left(t\right)\\ \;-a_{0}u\left(t\right)x_{0,ub}\left(t\right)-r_{0}x_{0,ub}\left(t\right), \end{array}$$
    (Equation 9) $$\frac{dx_{H,ub}\left(t\right)}{dt}=b_{0}v\left(t\right)x_{0,ub}\left(t\right)-b_{1}x_{H,ub}\left(t\right)-sx_{H,ub}\left(t\right)-r_{0}x_{H,ub}\left(t\right)$$
    with the following two quantities:
    (Equation 10) $$\bar{u}\equiv u\left(t\right)+x_{E,0}\left(t\right)+x_{E,ub}\left(t\right),$$
    (Equation 11) $$\bar{v}\equiv v\left(t\right)+x_{H,ub}\left(t\right).$$
    The variables and rate parameters in Equations 5, 6, 7, 8, 9, 10, and 11 are defined in Tables 1 and 2.
  b. As a sanity check, sum up Equations 5, 6, 7, 8, and 9 and obtain the time derivative of the total protein concentration:
    (Equation 12) $$\begin{array}{c} \frac{d}{dt}\left[x_{0}\left(t\right)+x_{E,0}\left(t\right)+x_{E,ub}\left(t\right)+x_{0,ub}\left(t\right)+x_{H,ub}\left(t\right)\right]\\ \;=g\left(t\right)-r_{0}\left[x_{E,ub}\left(t\right)+x_{0,ub}\left(t\right)+x_{H,ub}\left(t\right)\right]. \end{array}$$
    This result makes sense because *g*(*t*) represents the ultimate source of protein production and the variables with a common coefficient *r*_0_ are the concentrations of ubiquitinated proteins destined for degradation.
  c. Let *x*(*t*) denote the total protein concentration:
    (Equation 13) $$x\left(t\right)\equiv x_{0}\left(t\right)+x_{E,0}\left(t\right)+x_{E,ub}\left(t\right)+x_{0,ub}\left(t\right)+x_{H,ub}\left(t\right).$$
    Equation 12 is rewritten as
    (Equation 14) $$\frac{dx\left(t\right)}{dt}=g\left(t\right)-r\left(t\right)x\left(t\right),$$
    where *r*(*t*) is the protein degradation rate given by
    (Equation 15) $$r\left(t\right)\equiv r_{0}\left[x_{E,ub}\left(t\right)+x_{0,ub}\left(t\right)+x_{H,ub}\left(t\right)\right]/x\left(t\right).$$
  d. Be cautious about the implicit physical or biological constraints on the variables and parameters. In the example of our model, all the molecular concentrations should be nonnegative. That is,
    (Equation 16) $$\begin{array}{c} x_{0}\left(t\right)\geq 0,x_{E,0}\left(t\right)\geq 0,x_{E,ub}\left(t\right)\geq 0,x_{0,ub}\left(t\right)\geq 0,x_{H,ub}\left(t\right)\geq 0,\\ u\left(t\right)\geq 0,v\left(t\right)\geq 0. \end{array}$$
    Additionally, because the protein synthesis rate cannot be negative, the following inequality should be satisfied:
    (Equation 17) g(t)≥0.

4. Transform the ODEs to utilize the experimentally available information on relatively downstream variables in the ODE model.
  a. In the example of our model, the experimentally available data were the total concentration *x*(*t*) of a protein rather than those of the individual sub-forms of the protein or the protein synthesis rate at the upstream level. Although the experimental data usually represent the relative, but not absolute, molecular concentrations, the use of the relative concentration for *x*(*t*) in our model only changes the “unit” of concentrations without loss of generality. On the other hand, if multiple datasets of their own relative levels are incorporated into the model variables of the same dimensional quantities, the appropriate proportionality coefficients or conversion factors should be introduced for the sake of a unified scale.
  b. Plug the following relation (from Equation 13) in Equations 5 and 6:
    (Equation 18) $$x_{0}\left(t\right)=x\left(t\right)-\left[x_{E,0}\left(t\right)+x_{E,ub}\left(t\right)+x_{0,ub}\left(t\right)+x_{H,ub}\left(t\right)\right].$$
  c. *g*(*t*) in Equation 5 is rewritten as
    (Equation 19) $$g\left(t\right)=\frac{dx_{0}\left(t\right)}{dt}+a_{0}u\left(t\right)x_{0}\left(t\right)-a_{1}x_{E,0}\left(t\right)-sx_{H,ub}\left(t\right),$$
    where *x*_0_(*t*) is replaced by the right-hand side in Equation 18. We are now ready for the BS of our model, using Equations 6, 7, 8, 9, 10, 11, 18, and 19. Note that, in usual practice, *x*(*t*) and other variables are simulated using the profile of *g*(*t*); in our BS, *g*(*t*) and other variables are reversely simulated using the profile of *x*(*t*), through Equations 6, 7, 8, 9, 10, 11, 18, and 19.

5. Write the codes for simulation.
  a. Import Python modules (scipy).
  b. Read the time-course data of the total protein abundance. If the data do not span more than a single circadian period, expand them by the repetition of the data points to multiple circadian periods. This step is necessary if one wants to simulate the long-term asymptotic behavior of our circadian model, as will be explained later.
  c. Interpolate the data points of the total protein abundance. This step is necessary for the construction of a continuous trajectory of *x*(*t*) for our model simulation, given the discrete nature of time points with available data.
    i. Import interp1d module from scipy.interpolate.
    ii. Implement a cubic interpolation by setting the option “kind = ‘cubic’”. The result is an interpolated curve of *x*(*t*), as exemplified by Figure 2A.

**Table 1:** Parameters in the protein degradation model

Parameter	Meaning
*a*_0_	Rate of ubiquitin ligase binding to a substrate protein.
*a*_1_	Dissociation rate of a ubiquitin ligase and a not-ubiquitinated protein.
*a*_2_	Dissociation rate of a ubiquitin ligase and a ubiquitinated protein.
*b*_0_	Rate of deubiquitinating enzyme binding to a ubiquitinated protein.
*b*_1_	Dissociation rate of a deubiquitinating enzyme and a ubiquitinated protein.
*q*	Ubiquitination rate of a protein binding to a ubiquitin ligase.
*s*	Deubiquitination rate of a protein binding to a deubiquitinating enzyme, lumped with its subsequent dissociation from the deubiquitinating enzyme.
*r*_0_	Degradation rate of a ubiquitinated protein.
$\bar{u}$	Total ubiquitin ligase concentration.
$\bar{v}$	Total deubiquitinating enzyme concentration.

**Table 2:** Variables in the protein degradation model

Variable	Meaning
*t*	Time.
*x*_0_(*t*)	Concentration of a free protein without ubiquitination.
*x*_*E*,0_(*t*)	Concentration of a not-ubiquitinated protein that is binding to a ubiquitin ligase.
*x*_*E*,*ub*_(*t*)	Concentration of a ubiquitinated protein that is binding to a ubiquitin ligase.
*x*_0,*ub*_(*t*)	Concentration of a ubiquitinated protein that is not binding to a ubiquitin ligase.
*x*_*H*,*ub*_(*t*)	Concentration of a ubiquitinated protein that is binding to a deubiquitinating enzyme.
*g*(*t*)	Protein synthesis rate.
*u*(*t*)	Concentration of a free ubiquitin ligase.
*v*(*t*)	Concentration of a free deubiquitinating enzyme.
    iii. Build a Python function that uses time, an array of concentrations, an array of kinetic parameters, an interpolated curve of *x*(*t*) as input, and returns an array of the model variables and their time derivatives in Equations 6, 7, 8, 9, 10, 11, 18, and 19 as output.
  d. Solve the ODEs with scipy.solve_ivp module.
    i. Import solve_ivp module from scipy.integrate.
    ii. Set the parameters “fun”, “t_span”, “t_eval”, and “y0”. Here, “fun” is a Python function of which input is ‘t’ (time) and ‘y’ (an array of the values of variables) and output is an array of the time derivatives of the variables. “t_span” is a tuple containing two time points that indicate the beginning and end of the simulation. “t_eval” is an array of time points at which to store the computed solutions. “y0” is an array of the initial states of the variables.
    iii. When solving the system of ODEs numerically, we must take the following into consideration:(1) Selection of time step: When selecting the maximum time step for the simulation, it should be much smaller than the time scale of dynamics. For the circadian proteins, the time scale is ∼24 h. Therefore, it is safe to select the maximum time step much smaller than an hour. Moreover, checking whether the solution does not noticeably change with smaller time steps will assure the selection of an adequate time step. In the scipy.solve_ivp module, changing the “max_step” option would modify the maximum time step to solve the ODEs.(2) Stiffness of the problem: If the ODEs contain stiff terms, the Runge-Kutta method is not recommended. In this case, “LSODA”, “BDF”, or “Radau” methods are recommended. The method to solve the ODEs can be changed by setting the “method” option in the scipy.solve_ivp module.(3) Numerical error tolerance: When solving the system of ODEs, estimated errors can be controlled. “atol” and “rtol” options in the scipy.solve_ivp module can be used to control absolute and relative errors respectively.

6. Test the behaviors of the ODE model.
  a. Check whether the model output is not too sensitive to the initial conditions, given the profile of the observable (e.g., *x*(*t*) in our case) and the parameter values. This step is to determine whether the simulation outcome is essentially unique or not, regardless of particular initial conditions. One method is to randomly sample the initial conditions of each variable within a physiologically-relevant range and then check whether the simulation outcomes converge at similar trajectories. In the case of our model, the code in the following command allows the test of this initial condition dependency:
    > python check_initial_conditions_sensitivity.py
    The execution of the code gives the graphs of each variable as the functions of time with different initial conditions. Figures 2B and 2C demonstrate that *g*(*t*) and *r*(*t*) in our model converge well respectively, regardless of their initial conditions when *x*(*t*) (Figure 2A) and parameter values are assigned for the simulation.
  b. Determine the minimum simulation length to approach the asymptotic solutions of the ODE model. By running the simulation for a long enough time, i.e., setting the large values for ‘t_span’ and ‘t_eval’ options, check when the ODE solutions exhibit saturated and sustained oscillations in our case. If the simulation does not result in the stable oscillations, try longer simulation lengths. Identify the minimum simulation length to ensure the stable oscillations. The code in the following command allows this test:
    > python check_long_time.py
    Executing the code gives the graphs of each variable over a long time. Through these graphs, one can determine the minimum simulation length.
  c. Check whether the ODE solutions satisfy all the physical and biological constraints and thus can be considered as feasible solutions. Some ODE solutions may not satisfy these constraints, particularly in the case of BS. The reason is that BS does not run in a natural causal direction from upstream to downstream levels, but traces back the upstream states without the prospect of their compatibility with the parameter values. In other words, only the parameter values with the feasible solutions of BS are compatible with the downstream observable, and hence BS can identify those sensible parameter values. In the example of our model, the solutions should satisfy all the constraints in Equations 16 and 17. The code in the following command allows this feasibility test of the model solutions:
    > python check_physical_constraints.py
    The code verifies whether the constraints in Equations 16 and 17 are satisfied or not (Figure 2D).
  d. Debug the code of the ODE model. By simulating different parameter values, one can check the potential bugs in the code. In the example of our model, if $\bar{v}$ is set to 0, *x*_*H*,*ub*_(*t*) should be zero. In Equation 8, if *a*_0_*u*(*t*) and *b*_0_*v*(*t*) are much higher than *a*_2_ and *b*_1_ by setting *a*_0_, *b*_0_, $\bar{u}$, and $\bar{v}$ as relatively high and setting *a*_2_ and *b*_1_ as relatively low, then *x*_0,*ub*_(*t*) should become very small (Figures 2E and 2F). The code in the following command allows these bug tests:
    > python check_debugging.py
    Executing this code gives the parameter conditions and the graphs of the relevant simulation results.

### Sampling of parameter values

**Timing: 1 h to several days (depending on the size of parameter dimension)**

In this section, we will describe how to sample a large number of parameter values with multiple CPU cores. Utilizing parallel computing would save much of the simulation time.

7. Generate multiple parameter values and run the BS with these parameter values.
  a. If the number of the parameter values is too large, the Python modules “multiprocessing” and “mpi4py” can save much of the simulation time.
    i. How to use “multiprocessing” module: construct the wrapping module that only takes “parameter_sets”. The “parameter_sets” is a list of *N* parameter sets, and the wrapper is a wrapping module that takes a single parameter set as input. Parallel computing with a number of CPU cores can be implemented using these modules.
      > import multiprocessing as mp
      > parameter_sets = [parameter_set_1, …, parameter_set_N]
      > pool = mp.Pool(Number_of_CPU_cores)
      > pool.map(wrapper, parameter_sets)
    ii. How to use “mpi4py” module: “mpi4py” module allows the model simulation with each node. An example is shown below.
      > from mpi4py import MPI
      > comm = MPI.COMM_WORLD
      > num_processor = comm.Get_size()
      > rank = comm.Get_rank()
      > simulation_done_by_one = 0
      > simulation_target_by_one = N
      > while(simulation_done_by_one < simulation_target_by_one):
      > …
      > simulation_done_by_one += 1
      Here, N is the target number of the simulations in one node.
      ***Note:*** In both the cases i and ii above, be careful when writing a file. If two different nodes access the same file, this file may not be readable at the end.

## Expected outcomes

The BS method allows us to identify the valid parameter sets for experimentally available downstream data and to inspect the internal dynamics of the system with the fixed profiles of particular components. The latter would be useful for rigorous mechanistic inspection of a dynamical system. For example, we controlled for the protein profile *x*(*t*) in Lim et al. (2021) and found that the degradation rate *r*(*t*) tends to be more rhythmic with a lower level of a ubiquitin ligase $\bar{u}$. In addition, we identified a definite lower bound of $\bar{u}$ for the establishment of a given profile of *x*(*t*) itself, along with other interesting phenomena. Without the help of the BS, these clear conclusions may not be drawn, because in the conventional simulation with a fixed profile of the protein synthesis rate *g*(*t*), the change of $\bar{u}$ modifies the oscillatory form of *x*(*t*) itself and thus does not clearly separate the effect of $\bar{u}$ from that of the *x*(*t*) profile on the generation of rhythmic *r*(*t*) (Lim et al., 2021).

## Limitations

Our BS method is based on ODE models, and its applications beyond ODE models are not yet straightforward. In addition, it should be noticed that BS does not aim to infer unknown parameter values; rather, it infers the upstream dynamics with given parameter values, and thereby identifies the parameter values with feasible upstream states, compatible with the downstream observables. If the BS is implemented with randomly-sampled parameters, the large number of parameter sets may need to be sampled as the dimension of the parameter space increases ( *d*^*N*^ parameter sets, where *N* is the number of parameters in the model and *d* is the number of different values sampled for each parameter). Obviously, this massive parameter sampling can be computationally demanding.

## Troubleshooting

### Problem 1

When solving an ODE model with the scipy.solve_ivp module, the computation time may sometimes be very long (step 6 of section “application of BS to a circadian protein degradation model”).

### Potential solution

When the interpolated curve of the experimental profile is smooth enough, the option “method = “RK45”” and “method = “LSODA”” will not take much different computational times. However, if the interpolated curve is not smooth enough, “method = “RK45”” will take longer computational time. Therefore, in this case, we recommend the use of “method = “LSODA”” for shorter computational time.

### Problem 2

If the profile of *x*(*t*) in our model is too noisy, most BS results will not give the feasible solutions of the upstream states. These noisy patterns are likely to come from very high temporal resolution of the experimental data. For example, the PER2 profile for our BS in Lim et al. (2021) was obtained from the data of Zhou et al. (2015) with 6-min resolution. (step 2.a of section “BS in general cases” and step 4.a of section “application of BS to a circadian protein degradation model”).

### Potential solution

Time window averaging or other denoising techniques can be applied to the noisy profile. However, be cautious of the possibility that such smoothening may distort the original patterns in the profile.

### Problem 3

The ODE solutions are not sometimes accurate enough (step 5.d.iii and step 6 of section “application of BS to a circadian protein degradation model”).

### Potential solution

There is an option in scipy.solve_ivp to control the error-tolerance level in numerical integration of ODEs. By adjusting the options ‘atol’ and ‘rtol’, one can manage the absolute and relative levels of the tolerance to the numerical errors, respectively. However, too small ‘atol’ and ‘rtol’ values can considerably slow down the computation.

### Problem 4

When simulating excessively many parameter sets, it is difficult to manually determine whether ODE solutions have reached their attractors or not within the simulation time (step 7 of section “sampling of parameter values”).

### Potential solution

Some parameter sets may take long computation time towards the asymptotic states of the model outcome. In the case of our model, if the peak values of the oscillating variables decrease or increase with more than some fold change in the past two circadian periods, these variables may not be considered to reach the stable oscillatory states at that time.

### Problem 5

When solving the system of ODEs with the scipy.solve_ivp module, the solution includes time points assigned in the “t_eval” argument. However, a continuous time series of the solution might be needed in some cases (step 5.d of section “application of BS to a circadian protein degradation model”).

### Potential solution

If an option in the solve_ivp module, “dense_output” is set to “True”, it will return a class instance for the ODE solution at a given time point. However, this option might increase the computation time for the solution especially when the solution involves a long time series. Alternatively, the use of small time steps for the “t_eval” option and the interpolation of the solution over the last one or two time periods only would save the computation time.

## Resource availability

### Lead contact

Further information and requests for resources and reagents should be directed to and will be fulfilled by the lead contact, Pan-Jun Kim (panjunkim@hkbu.edu.hk).

### Materials availability

This study did not generate any unique reagents.

## Data Availability

This study did not generate new experimental data. Source codes for our model simulation have been deposited to public repository GitHub, and the link is provided in the key resources table.
